# Bioprospecting for Novel Bacterial Sources of Hydrolytic Enzymes and Antimicrobials in the Romanian Littoral Zone of the Black Sea

**DOI:** 10.3390/microorganisms10122468

**Published:** 2022-12-14

**Authors:** Robert Ruginescu, Paris Lavin, Lavinia Iancu, Selma Menabit, Cristina Purcarea

**Affiliations:** 1 Department of Microbiology, Institute of Biology Bucharest of the Romanian Academy, 296 Splaiul Independentei, 060031 Bucharest, Romania; 2 Departamento de Biotecnología, Facultad de Ciencias del Mar y Recursos Biológicos, Universidad de Antofagasta, 601 Angamos Av., Antofagasta 1240000, Chile; 3 Forensic Science Program, Department of Criminal Justice, University of North Dakota, Grand Forks, ND 58202, USA; 4 National Institute for Research and Development on Marine Geology and Geoecology-GeoEcoMar, 024053 Bucharest, Romania

**Keywords:** marine bacteria, seawater microbial diversity, Illumina sequencing, halophilic bacteria, extracellular hydrolases, lipase, carbohydrate-degrading enzymes, antimicrobial activity

## Abstract

Marine microorganisms have evolved a large variety of metabolites and biochemical processes, providing great opportunities for biotechnologies. In the search for new hydrolytic enzymes and antimicrobial compounds with enhanced characteristics, the current study explored the diversity of cultured and uncultured marine bacteria in Black Sea water from two locations along the Romanian coastline. Microbial cell density in the investigated samples varied between 65 and 12.7 × 10^3^ CFU·mL^−1^. The total bacterial community identified by Illumina sequencing of 16S rRNA gene comprised 185 genera belonging to 46 classes, mainly Gammaproteobacteria, Alphaproteobacteria, Flavobacteriia, and 24 phyla. The 66 bacterial strains isolated on seawater-based culture media belonged to 33 genera and showed variable growth temperatures, growth rates, and salt tolerance. A great fraction of these strains, including *Pseudoalteromonas* and *Flavobacterium* species, produced extracellular proteases, lipases, and carbohydrases, while two strains belonging to the genera *Aquimarina* and *Streptomyces* exhibited antimicrobial activity against human pathogenic bacteria. This study led to a broader view on the diversity of microbial communities in the Black Sea, and provided new marine strains with hydrolytic and antimicrobial capabilities that may be exploited in industrial and pharmaceutical applications.

## 1. Introduction

Covering nearly three quarters of the Earth’s surface, oceans and seas emerge as the largest reservoirs of biodiversity [1]. Among the great diversity of marine life forms, microorganisms are highly adapted to different physical, chemical, and biological conditions and thus occur in all the varied habitats of the marine environment. They are found as members of the plankton, attached to the surfaces of inanimate structures and living organisms in deep marine sediments and subsurface rocks, as well as in more extreme habitats such as hydrothermal vents, deep hypersaline anoxic basins, and sea ice [1]. To thrive in such a wide range of habitats, marine microorganisms have evolved a large diversity of metabolic pathways by which they not only ensure their own survival but also maintain the web of life in the marine environment [1,2].

Besides their ecological roles, marine microorganisms provide great opportunities for biotechnological exploitation as sources of enzymes (e.g., hydrolases, DNA polymerases, and oxidoreductases), bioactive molecules (e.g., antimicrobials and nutraceuticals), polymers (e.g., polysaccharides and polyhydroxyalkanoates), and biofuels (e.g., microalgae) which can be used in industries, medicine, and research [1,3,4,5]. Among the numerous marine-derived products, enzymes feature prominently due to their continuously increasing demand on the global market. For instance, hydrolases that degrade proteins, lipids, and carbohydrates dominate the global market for industrial enzymes, which is expected to reach USD 8.7 billion by 2026 [6].

The remarkable diversity of marine microorganisms is reflected in the wide variety of enzymes with habitat-related chemical and stereochemical characteristics, such as salt tolerance, hyperthermostability, cold adaptability, barophilicity, chemoselectivity, regioselectivity, and stereoselectivity [3,7]. Accordingly, many bioprospecting studies based on both culture-dependent and metagenomic approaches are currently aiming to discover new marine microorganisms-derived enzymes with unique properties to improve existing bioprocesses and develop novel biotechnologies [8,9,10]. Among these, extracellular proteases, lipases, and carbohydrate-degrading enzymes that are stable over a wide range of temperatures, salt concentrations, pH values, as well as in the presence of metal ions, bleaching chemicals and surfactants produced by marine bacteria belonging to different taxonomic groups such as *Staphylothermus marinus*, *Vibrio* spp., *Bacillus* spp., *Psychrobacter* spp., and *Pseudoalteromonas* spp. are of particular importance for the food, detergent, and biofuel industries [7,11,12]. Moreover, the high substrate specificity and enantioselectivity of enzymes produced by marine species, such as L-asparaginase and L-glutaminase from *Bacillus* spp., are crucial for therapeutic applications [3].

Among the marine microorganisms-derived metabolites, the compounds with antimicrobial activity against human pathogenic species are of high interest taking into consideration that numerous antimicrobial drugs obtained from terrestrial fungi (e.g., penicillin) and bacteria (e.g., tetracyclines, aminoglycosides, and macrolides) are becoming less effective as a consequence of their overuse [1]. Over the past decades, studies conducted in various marine habitats around the world reported thousands of secondary metabolites with antimicrobial effects, particularly non-ribosomal peptides and polyketides mainly from species belonging to phyla Actinomycetota, Bacillota, and Pseudomonadota. However, only a few were approved as drugs (e.g., cephalosporin, anthracimycin, marinopyrrole A) or are in pre-/clinical trials [13,14,15,16]. Consequently, the current trend is focused on bioprospecting untapped habitats for sources of novel metabolites with antimicrobial activity against multidrug-resistant pathogens, one of the greatest threats to human health [13].

In this respect, the Black Sea represents a yet underexplored environment regarding the biotechnological potential of its microbiota. In contrast to most seas, the Black Sea is characterized by an oxygenated and brackish surface layer of river origin overlying an euxinic and saline deep layer (>150 m) of Mediterranean origin [17]. The taxonomic and metabolic profiles of the Black Sea microbial communities were determined at different locations, depths, and time points by culture-independent studies, with particular interest on the biological processes that occur in the euxinic water layer and at the oxic/anoxic interface [18,19,20,21,22,23,24]. The great effort to elucidate the ecological roles of microorganisms inhabiting this unique environment was, however, counterbalanced by a lack of studies aiming to explore their biotechnological potential. To our knowledge, relatively few investigations carried out in the Black Sea have targeted the discovery of novel valuable metabolites such as enzymes [25,26] and bioactive molecules [27,28].

In this context, the current study reported the diversity of the uncultured bacterial community from the Romanian littoral zone of the Black Sea using 16S rRNA gene Illumina sequencing, the isolation and identification of 66 aerobic chemo-organo-heterotrophic bacterial strains, as well as the characterization of their extracellular hydrolytic and antimicrobial profiles. These data represent a first step in identifying new valuable industrial and pharmaceutical compounds, and provide a broader view of the yet uncultured microbial taxa for further exploring their applicative potential.

## 2. Materials and Methods

### 2.1. Site Description, Sample Collection and Processing

The Black Sea is a large inland body of water connected to the Mediterranean basin by the Bosphorus Strait. It is located between Southeastern Europe and Western Asia and is bordered by six countries, including Romania. The Romanian coastline stretches for 244 km in the western part of the basin, between the Danube Delta (in the north) and the Bulgarian border (in the south) [29].

**Figure 1:**
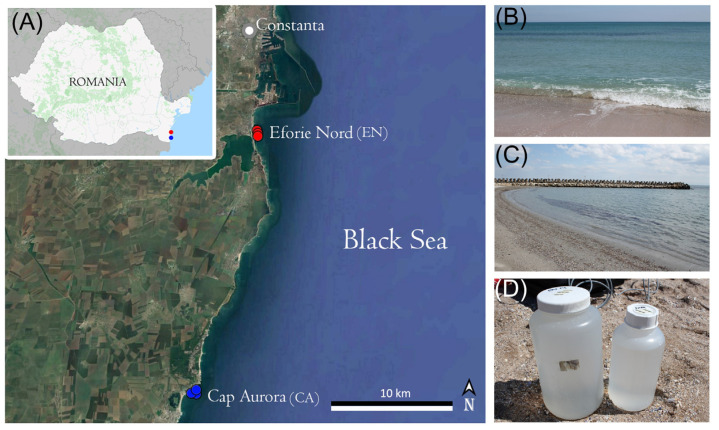
Black Sea sampling. (**A**) Satellite image (obtained with Google Earth Pro) showing the geographic locations of the sampling sites; (**B**) EN sampling location; (**C**) CA sampling location; (**D**) Collected water samples. The photos (**B**–**D**) were taken by L. Iancu.

Water samples were collected in April 2021 from two locations of the Romanian Black Sea seashore, i.e., Eforie Nord (EN) and Cap Aurora (CA). EN (44°3′33.03912′′ N, 28°38′28.64356′′ E) is among the most popular resorts in the area, and is located about 9 km from Port Constanta, thereby being more affected by anthropic pollution compared with CA (43°50′53.2′′ N, 28°36′14.4′′ E) which is located 27 km south of EN (Figure 1A). Moreover, the coastline in EN is more open and exposed to waves (Figure 1B) compared with CA which has many lagoon-like areas (Figure 1C). For each location, three sampling sites were selected at 2–3 m from the seashore and at about 0.2 m below the water surface. Water samples were collected in sterile plastic bottles (Figure 1D) and transported to the laboratory under a constant temperature of 10 °C.

Samples were processed 18 h after collection. A volume of 0.75–0.9 L of each seawater sample was filtered under aseptic conditions through 0.22 µM sterile MF-Millipore membrane filters (Merck, Darmstadt, Germany), and the resulted biomass was further stored at −20 °C until total DNA extraction for Illumina sequencing of the 16S rRNA gene. The resulting filtered seawater was stored in the dark at 20 °C until used for growth media preparation. The unfiltered seawater samples were used as inocula for cultivating marine bacteria (see Section 2.3).

### 2.2. Physicochemical Parameters Measurement

In situ measurements of the seawater physicochemical parameters (temperature, dissolved oxygen, and salinity) were carried out with a portable multiparameter (Model HI98194, Hanna Instruments, Woonsocket, RI, USA). The pH of water samples was measured in the laboratory at 23 °C using a pH meter (Isolab, Eschau, Germany).

### 2.3. Isolation and Identification of Marine Bacterial Strains

Water samples were serially diluted in sterile saline solution (0.9% NaCl, *w*/*v*) and aliquots (1 mL) were pour-plated onto marine agar (BD Difco, Franklin Lakes, NJ, USA) and enriched seawater agar media suitable for culturing marine bacteria. The latter was prepared by dissolving 5 g peptone (Oxoid, Basingstoke, UK), 1 g yeast extract (BD Difco), 1 g glucose monohydrate (Merck, Darmstadt, Germany) and 17 g agar (Scharlau, Barcelona, Spain) in 1 L of previously filtered seawater. The pH was adjusted to 7.2 before autoclaving.

Plates were incubated at 28 °C for 7 days and the number of bacterial colonies was used to calculate the cell density expressed as colony-forming units (CFUs) per mL of seawater. Colonies that displayed distinct morphologies were further purified by re-streaking onto the same growth media used for cultivation.

The isolated bacterial strains were taxonomically identified by PCR amplification and sequencing of the 16S rRNA gene. Based on the established growth characteristics, the strains were cultivated in enriched seawater broth medium at 30 °C for 48–72 h, and cells contained in 1 mL of culture were harvested by centrifugation for 10 min at 6000× *g*. Genomic DNA was extracted using a DNeasy Blood and Tissue Kit (Qiagen, Hilden, Germany) following the standard protocol for bacteria. PCR amplification of the 16S rRNA gene was carried out as previously described [30], using primers 27F (AGAGTTTGATCCTGGCTCAG) and 1492R (GGTTACCTTGTTACGACTT) [31]. Amplicons were purified using a QIAquick PCR Purification Kit (Qiagen, Hilden, Germany) and sequenced using the amplification primers (Macrogen Europe B.V., Amsterdam, The Netherlands). The raw DNA sequences were analyzed using the CodonCode Aligner software (v.9.0.2) and the low-quality bases from the ends of sequences were trimmed. The resulted sequences were compared with those available in the GenBank database (NCBI) using BLASTN [32].

### 2.4. Bacterial Growth Characterization

#### 2.4.1. Halophily and Halotolerance Assessment

The salt requirement and tolerance of the bacterial isolates were investigated by growing on solid media containing 0–25% (*w*/*v*) salts. The growth medium without salts contained 5 g peptone (Oxoid), 1 g yeast extract (BD Difco), and 17 g agar (Scharlau) in 1 L distilled water. The medium with 2.92% salts was prepared by adding the corresponding amount of NaCl to the composition mentioned above. The medium with 3.4% salts was represented by marine agar (BD Difco), while the media with 7–25% salts were prepared by supplementing the marine agar medium with the corresponding amount of NaCl. The strains were spot-inoculated in duplicate using fresh solid inocula, and the growth was monitored at 30 °C up to seven days. The optimal salt concentrations corresponded to the ones sustaining the fastest growth. The strains were categorized as halotolerant, slightly halophilic, or moderately halophilic according to Kushner’s classification [33].

#### 2.4.2. Growth Temperature Assessment

The growth temperature interval for the bacterial isolates was determined by culturing at 4, 10, 15, 20, 30, 40, 50, 55, and 60 °C on marine agar medium. The strains were spot-inoculated in duplicate using fresh solid inocula and growth was monitored daily for seven days. Under these conditions, the estimated optimal growth temperature corresponded to the fastest colonies formation.

#### 2.4.3. Growth Rate Determination

The growth rate of the bacterial strains was monitored spectrophotometrically (OD_620_) by cultivation at 30 °C for 72 h in 96-well F-Bottom microplates (Greiner Bio-One, Kremsmünster, Austria) using a FLUOstar Omega spectrophotometer (BMG Labtech, Ortenberg, Germany). Each well contained 200 μL enriched seawater medium and 4 μL fresh inoculum with OD_620_ of 0.2. Prior to each cycle, plates were stirred for 5 min at 200 rpm. All experiments were performed in triplicate. Based on the values recorded, growth curves were generated using Microsoft Excel. For each bacterial strain, the values corresponding to the logarithmic growth phase were entered in Cell Calculator++ program [34] to calculate the growth rate and doubling time (DT).

### 2.5. Functional Characterization of Bacterial Strains

#### 2.5.1. Extracellular Hydrolytic Activities

The screening for extracellular hydrolytic activities (i.e., protease, lipase, amylase, cellulase, xylanase, and pectinase) was carried out by culturing the bacterial strains on enriched seawater agar supplemented with one of the following substrates at indicated concentrations (g·L^−1^): casein (10), Tween-80 (10), starch (10), carboxymethyl cellulose (CMC) (5), xylan (10), or pectin (10). The strains were spot-inoculated onto the surface of agar plates using fresh solid inocula and incubated at 30 °C for 7 days. Hydrolytic activities were indicated by a clear/opaque zone around the colonies [30]. The experiments were performed in duplicate. The levels of enzyme activity (LEA) were evaluated based on the diameter of the hydrolysis zone divided by the diameter of the bacterial colony in millimeters [30], and categorized as high (LEA > 3), medium (LEA 2–3), or low (LEA < 2).

#### 2.5.2. Antimicrobial Activities

Production of antimicrobial compounds by the marine strains was assayed using the soft-agar overlay technique [35]. The bacterial strains were spot-inoculated onto the surface of marine agar plates and incubated at 20 °C for 4 days. Subsequently, the plates were overlaid with soft Mueller–Hinton agar (0.7% agar) premixed with 10^7^ CFU of the indicator strain and incubated at 35 °C for 24 h. A clear zone around the culture spots indicated the antagonistic activity against the indicator strains. When the soft-agar overlay technique did not show accurate results, the antibacterial activities were retested using the cross-streak method [36]. The indicator strains were represented by the human pathogens *Escherichia coli* ATCC 25922, *Staphylococcus aureus* ATCC 25923, *Pseudomonas aeruginosa* ATCC 15442, *Listeria monocytogenes* ATCC 1911, and *Salmonella enterica* subsp. *enterica* serovar Typhimurium ATCC 14028.

The marine strains showing positive results were subsequently screened against nine clinical isolates (see Section 3.5.2) provided by the Research Institute of the University of Bucharest following the same protocol. All tests were performed in duplicate.

### 2.6. Bacterial Community Composition Assessment

#### 2.6.1. Total DNA Extraction and Illumina Sequencing of 16S rRNA Amplicons

The genomic DNA was extracted from the filters containing microbial biomass using a DNeasy Blood and Tissue Kit (Qiagen, Hilden, Germany) in accordance with a modified protocol described by Djurhuus et al. [37] that included an initial 12 min bead-beating cell disrupting step in innuSPEED Lysis Tubes X (Analitik Jena, Jena, Germany) using a SpeedMill PLUS Cell Homogenizer (Analitik Jena, Jena, Germany). Library construction and sequencing were performed by Macrogen (Seoul, South Korea). PCR amplification of the V3–V4 region of the 16S rRNA genes was carried out with the 341F/805R primer pair [38]. The DNA libraries were sequenced using an Illumina MiSeq 300PE platform.

#### 2.6.2. Sequence Analyses

The raw sequences were analyzed using dada2 package (v1.8) implemented in R (v4.0.2) [39]. Forward and reverse primers were removed using cutadapt (v4.1) [40], then sequences were trimmed and filtered (truncLen  =  c(230, 160), maxN = 0, maxEE = c(2, 2), truncQ = 2, rm.phix = TRUE, compress = TRUE, multithread = TRUE). Amplicon Sequence Variants (ASVs) were inferred from de-replicated sequences and chimeras removed using the “consensus” removal method. Taxonomic assignment of the ASVs was performed using the Silva 16S rRNA database (silva.nr.v138). The community analysis was performed in R environment with the phyloseq package (v1.40.0) [41].

### 2.7. Nucleotide Sequence Accession Numbers

The partial 16S rRNA gene sequences of cultured bacterial strains were deposited in GenBank (NCBI) under the accession numbers: OL672332–OL672377, OL662943–OL662984, and ON382270 (Supplementary Table S1). The raw Illumina-derived sequences were deposited in the NCBI Sequence Read Archive under the BioProject: PRJNA875633.

## 3. Results

### 3.1. Physicochemical Characteristics of Seawater

The physicochemical properties of Black Sea water from the Eforie Nord (EN) and Cap Aurora (CA) sampling sites showed similar values, except for the concentration of dissolved oxygen that was 2-fold higher in the CA area compared with EN (Table 1). This result may be attributed to the gulf coast topography of the CA area, which was less exposed to marine currents and characterized by a bloom of macroscopic algae near the seashore. The slightly alkaline pH (8.1) and the relatively low salinity (18 g·kg^−1^) from both locations were in agreement with the values measured previously in the surface water layer from various areas of the Black Sea [42]. In comparison with the Mediterranean Sea, which is characterized by an overall higher salinity (38 g·kg^−1^) due to the negative water balance [43], the Black Sea has a positive hydric balance that is responsible for maintaining its waters within the brackish category [17].

### 3.2. Abundance of Cultured Marine Bacteria

**Figure 2:**
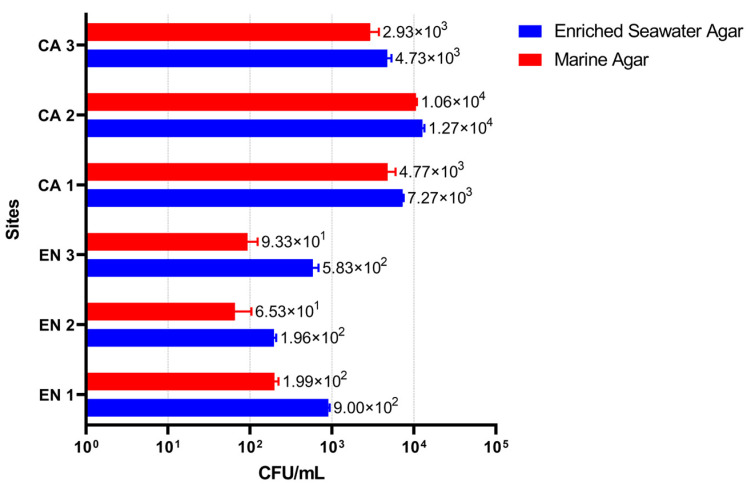
Microbial abundance of the cultured marine bacteria from the Black Sea. The number of colony-forming units (CFUs) per mL of water was calculated for the Cap Aurora (CA) and Eforie Nord (EN) sites as indicated in Methods. Error bars show the variations for three technical replicates.

The abundance of cultured bacteria from the analyzed Black Sea samples on marine water-based media varied considerably among the different investigated sites (Figure 2). The water samples collected from CA contained higher fractions of cultivable bacteria (2.93 × 10^3^–12.7 × 10^3^ CFU·mL^−1^) compared with those from EN (65–9 × 10^2^ CFU·mL^−1^), probably because the sampling sites in CA were located in a relatively stagnant water area characterized by reduced dispersion rates of bacterial cells. These values were comparable with bacterial cell densities reported in the Turkish littoral zone of the Black Sea (10^2^–10^7^ CFU·mL^−1^) [44] and in coastal areas of the Mediterranean Sea (10^3^–10^5^ CFU·mL^−1^) [45].

Moreover, the enriched seawater agar medium supported the growth of more CFUs than the marine agar, with a more pronounced difference (3–6-fold) for microbial cultures from the EN sites compared with the CA sites (up to 1.6-fold) (Figure 2). In this case, the high content of salts (3.4%) of the marine agar medium, representing a concentration almost double than that of the Black Sea water, can be responsible for inhibiting the growth of halosensitive species.

### 3.3. Diversity and Taxonomic Profile of the Total Bacterial Community from Black Sea Water

The bacterial diversity and community structure of the Romanian Black Sea coastline water were determined using a metagenomic approach based on 16S rRNA gene Illumina sequencing of duplicate seawater samples (i.e., EN1 and EN2) collected from Eforie Nord area. A total of 581,760 reads were obtained, corresponding to 34,750 raw sequences assigned to 2345 unique ASVs (Supplementary Table S2). Among these, 378 ASVs (16.1%) were present in both investigated sites, corresponding to the most abundant sequences (50.2% of the total number). Meanwhile, 950 and 1017 unique ASVs were found in EN1 and EN2, respectively (Supplementary Figure S1A). Alpha diversity analysis (Supplementary Table S2) indicated similar ecological indices values, corresponding to a relatively high Shannon–Weaver and Simpson indices indicating greater bacterial diversity with no major change between samples. The bacterial richness (S and Chao1) was high in both sampling sites with a difference of 67 ASVs (2.8%).

**Figure 3:**
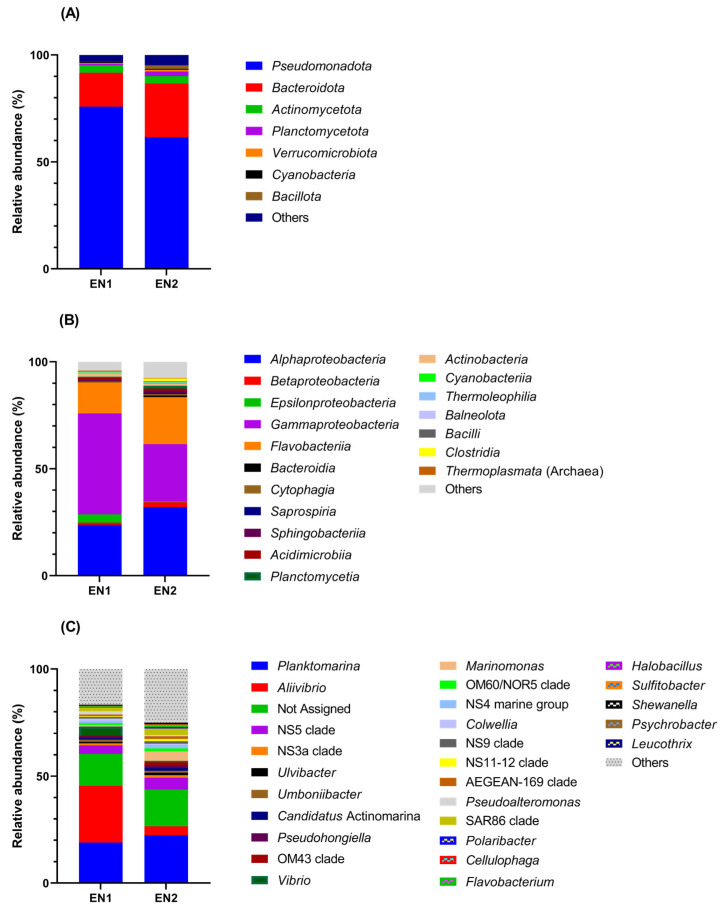
Community structure of uncultured bacteria from EN Black Sea sites. Relative abundances of the taxa at phylum (**A**), class (**B**), and genus (**C**) levels were determined as indicated in Methods.

Taxonomic assignment of the determined 16S rDNA ASVs highlighted the presence of a complex bacterial community composed of 185 genera belonging to 46 classes and 24 phyla (Figure 3). Although most taxa were common to both investigated sites, a higher number of unique taxa was found in EN2. Thus, in addition to 95 shared genera, 33 and 57 unique ones were identified in EN1 and EN2, respectively (Supplementary Figure S1B).

Analysis of the community structure from the Black Sea water samples revealed the dominance of taxa belonging to phylum Pseudomonadota (relative abundance of 68.6%), and a notable presence of Bacteroidota (20.5%), while other phyla such as Actinomycetota and Bacillota accounted for only 3.4% and 0.84%, respectively (Figure 3A). At class level (Figure 3B), a high contribution was attributed to Gammaproteobacteria (36.9%), Alphaproteobacteria (27.8%), and Flavobacteriia (18.3%), while other classes commonly found in marine environments such as Cyanobacteria, Betaproteobacteria, and Epsilonproteobacteria accounted for less than 2% relative abundance. At genus level, *Planktomarina* constituted an important fraction in both EN1 (18.9%) and EN2 (22.4%) sites, and *Aliivibrio* represented the prevalent genus in EN1, accounting for 26% of the uncultured bacterial community. Other identified genera and yet-unclassified marine clades (e.g., NS5 and NS3a from Flavobacteriaceae family) were scarcely represented by a relative abundance score below 4.7% (Figure 3C).

### 3.4. Taxonomic Diversity and Growth Characteristics of Cultured Bacterial Strains

**Figure 4:**
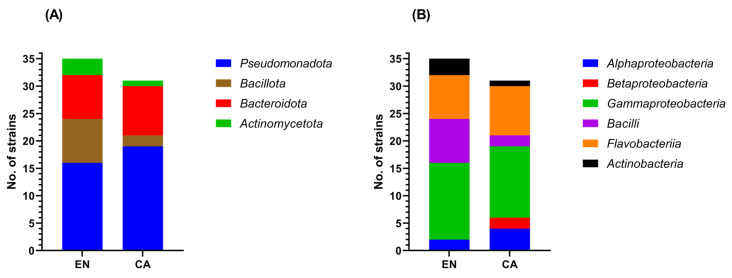
Taxonomic distribution of the cultured bacterial strains, retrieved from the EN and CA locations, at phylum (**A**) and class (**B**) levels.

Inoculation of water samples collected from the two Black Sea locations on marine water-based growth media and cultivation for 7 days at 28 °C led to the isolation of 89 bacterial colonies with apparently distinct morphology. These isolates were identified by 16S rRNA gene sequencing, resulting in 66 distinct strains based on the identity score with homologous bacteria from public databases (Supplementary Table S1). These strains were taxonomically assigned to 4 phyla (Figure 4A), 6 classes (Figure 4B), and 33 genera.

Among these strains, 27 (40.9%) were assigned to 10 genera of the class Gammaproteobacteria, including *Shewanella*, *Pseudomonas*, *Pseudoalteromonas*, *Paraglaciecola*, *Psychrobacter*, *Marinomonas*, *Marinobacter*, *Leucothrix*, *Granulosicoccus*, and *Enterovibrio*. The second most represented class was Flavobacteriia, counting 17 strains (25.8%) that belonged to the genera *Aquimarina*, *Algibacter*, *Cellulophaga*, *Flavobacterium*, *Maribacter*, *Polaribacter*, *Wenyingzhuangia*, and *Zobellia*. Within the class Bacilli, the 10 retrieved strains (15.1%) were from the genera *Bacillus*, *Halobacillus*, *Jeotgalibacillus*, *Metabacillus*, *Peribacillus*, and *Salinicoccus*. The class Alphaproteobacteria was represented by six strains (9.1%) from four genera (*Ahrensia*, *Sulfitobacter*, *Litoreibacter*, and *Roseobacter*). The class Actinobacteria included four strains (6.1%) belonging to the genera *Isoptericola*, *Micrococcus*, *Salinibacterium*, and *Streptomyces*, while the class Betaproteobacteria comprised two strains (3%) related to members of the genus *Hydrogenophaga* (Supplementary Table S1).

Regarding the origin of the 66 bacterial strains, 35 were isolated from EN and 31 from CA. Among these, 45 strains (68.2%) belonging to 15 genera were found in both locations, while 21 strains (31.8%) belonging to 18 genera were isolated exclusively from EN (11 strains; 9 genera) or CA (10 strains; 9 genera) (Supplementary Figure S2). At class level, members of the Alphaproteobacteria, Gammaproteobacteria, Flavobacteriia, Bacilli, and Actinobacteria were recovered from both sampling locations, while representatives of the Betaproteobacteria were isolated only from CA (Figure 4B).

**Figure 5:**
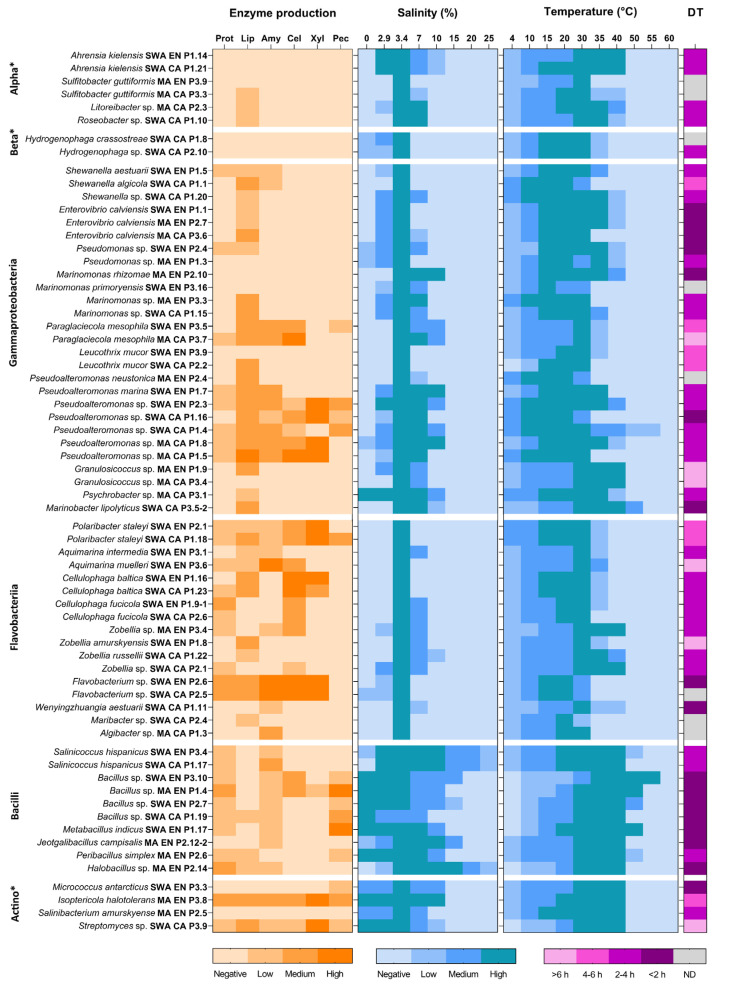
Heat maps of hydrolytic enzyme production and growth characteristics of the isolated bacterial strains. The levels of enzyme activity, salinity (% NaCl, *w*/*v*) and temperature (°C) intervals, and doubling time (DT) were determined as indicated in Methods. Alphaproteobacteria (*Alpha), Betaproteobacteria (*Beta), Actinobacteria (*Actino); not determined (ND).

Characterization of growth conditions (salinity, temperature, and doubling time) highlighted the functional diversity of the retrieved bacterial strain collection. The halotolerance profile of the isolated bacteria (Figure 5) revealed the halophilic nature of the high majority of the Black Sea strains, 48 (72.7%) requiring the presence of at least 2.9% or 3.4% salts for growth. Among these, 45 were slightly halophilic (grew optimally with 3.4% salts) and three strains (i.e., *Salinicoccus hispanicus* SWA CA P1.17, SWA EN P3.4, and *Halobacillus* sp. MA EN P2.14) which tolerated up to 25% salts were moderately halophilic. Moreover, 18 strains (27.3%) that did not require the presence of salts for growth, but tolerated concentrations of NaCl up to 7% and 15%, were halotolerant and extremely halotolerant, respectively. Most of the strains with the highest salt tolerance belonged to the class Bacilli (Figure 5).

The growth temperature interval of the recovered marine bacteria was also variable (Figure 5), with the highest number of strains (25) able to grow in the 4–40 °C interval, and others in a more restrained range of 4–35 °C (16 strains) and 4–30 °C (13 strains). At 4 °C, the majority of the isolates grew slowly (>3–4 days), and eight strains required incubation temperatures above 10 °C. A limited number of strains, mainly belonging to the classes Bacilli and Actinobacteria, could grow at temperatures up to 50 °C (6 strains) and 55 °C (2 strains) (Figure 5).

The doubling time (DT) calculated from the growth curves at 30 °C in enriched seawater medium was also variable, with most of the strains characterized by a DT < 2 h (17 strains) or within the 2–4 h interval (28 strains) (Figure 5). Marine bacteria showing the fastest growth rates belonged to the genera *Bacillus*, *Metabacillus*, *Jeotgalibacillus*, *Halobacillus*, *Micrococcus*, *Enterovibrio*, *Pseudomonas*, *Marinomonas*, *Pseudoalteromonas*, *Marinobacter*, *Flavobacterium*, and *Wenyingzhuangia*. Meanwhile, seven strains belonging to the genera *Shewanella*, *Paraglaciecola*, *Leucothrix*, *Polaribacter*, and *Isoptericola* had DT comprised between 4 and 6 h, and six strains of the genera *Paraglaciecola*, *Granulosicoccus*, *Aquimarina*, *Zobellia*, and *Streptomyces* showed DT higher than 6 h (Figure 5). However, it was not possible to determine the doubling time of eight strains due to their inability to grow under static conditions in 96-well microplates.

### 3.5. Functional Characteristics of Cultured Bacterial Strains

#### 3.5.1. Production of Extracellular Hydrolases

**Figure 6:**
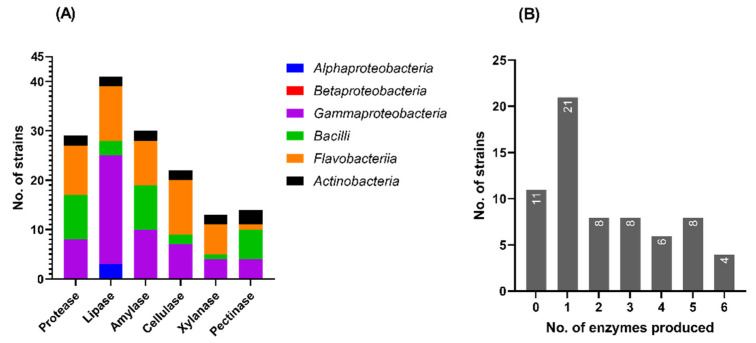
Hydrolytic activity profile of the Black Sea bacterial isolates. (**A**) Taxonomic distribution of different hydrolytic enzyme production; (**B**) distribution of strains exhibiting a particular number of extracellular hydrolases.

Among the 66 bacterial strains that were screened for the production of extracellular enzymes able to degrade proteins (i.e., casein), lipids (i.e., Tween 80), and polysaccharides (i.e., starch, CMC, xylan, and pectin), 55 (83%) exhibited at least one hydrolytic activity (Supplementary Figure S3). Lipase activity was found for most of the marine isolates (41), followed by amylases and proteases produced by 30 and 29 strains, respectively. Only a third of the strains (22) were able to produce cellulases, while a lower number presented pectinase (14 strains) and xylanase (13 strains) activities (Figure 6A). There were no major differences in the extracellular hydrolytic activity profiles with respect to the locations (EN or CA) of the isolated strains (Supplementary Figure S4). A third of the strains (21) presented single hydrolytic activities (generally lipolytic), while half (34 strains) produced combinations of two or more enzymes (Figure 6B).

The taxonomic distribution of the hydrolytic enzyme production by the marine bacterial isolates revealed a class-dependent preference for most of the tested enzymes (Figure 6A). Thus, most of the Gammaproteobacteria strains (81.5%) synthesized lipases. Within the class Flavobacteriia, the strains were able to produce mainly lipase (64.7%), cellulase (64.7%), protease (58.8%), and amylase (52.9%). Moreover, the strains assigned to the class Bacilli showed mainly proteolytic (90%), amylolytic (90%), and pectinolytic (60%) activities, while 50% of the Actinobacteria strains produced all the six hydrolases. Meanwhile, the hydrolytic potential of the Alphaproteobacteria strains was quite limited, the only detected extracellular activity of three strains (50%) being the lipolytic one against Tween 80. Betaproteobacteria strains did not show any of the six hydrolytic activities tested (Figure 6A; Supplementary Table S3). The scarce hydrolytic enzyme repertoire produced by Alphaproteobacteria and Betaproteobacteria strains can be attributed to their inherent inability to secrete enzymes extracellularly. In this respect, a previous study carried out by meta-omics analyses on seawater from the Pacific, Atlantic and Southern Ocean identified Gammaproteobacteria and Bateroidota as the main (>75%) contributors to the pool of extracellular carbohydrate-degrading enzymes and peptidases, while Alphaproteobacteria showed an important contribution to the pool of intracellular hydrolases [46].

The highest hydrolytic activities were identified in the case of *Pseudoalteromonas*, *Paraglaciecola*, *Polaribacter*, *Aquimarina*, *Cellulophaga*, *Flavobacterium*, *Bacillus*, *Metabacillus*, *Isoptericola*, and *Streptomyces* species (Figure 5). Thus, *Pseudoalteromonas* sp. strains SWA EN P2.3, SWA CA P1.16, MA CA P1.8, and MA CA P1.5 showed five or six hydrolytic activities, among which the highest (LEA > 3) were xylanolytic, cellulolytic and lipolytic. The SWA EN P2.6 and SWA CA P2.5 strains belonging to *Flavobacterium* sp. were able to produce five hydrolases with medium or high LEA. *Paraglaciecola mesophila* SWA EN P3.5 and MA CA P3.7 produced cellulase, amylase and lipase with medium or high LEA (Figure 5). *Polaribacter staleyi* (SWA EN P2.1 and SWA CA P1.18) and *Cellulophaga baltica* (SWA EN P1.16 and SWA CA P1.23) strains produced xylanase and cellulase with medium or high LEA. *Aquimarina muelleri* SWA EN P3.6 exhibited the highest amylolytic activity among the isolated strains. *Bacillus* sp. MA EN P1.4 and *Metabacillus* indicus SWA EN P1.17 showed high pectinolytic activities, and *Isoptericola halotolerans* MA EN P3.8 had the ability to produce all six enzymes with medium or high LEA. Similarly, *Streptomyces* sp. SWA CA P3.9 synthesized all six hydrolases, but only lipase and xylanase showed medium and high LEA, respectively (Figure 5).

#### 3.5.2. Production of Antimicrobial Compounds

Screening for the production of antimicrobial compounds by the bacterial isolates from the Black Sea water against five human pathogens led to the identification of two strains (i.e., *Aquimarina muelleri* SWA EN P3.6 and *Streptomyces* sp. SWA CA P3.9) showing an inhibitory effect against *S*. *aureus* ATCC 25923 and *L. monocytogenes* ATCC 1911 (Table 2). Further tests for their putative antimicrobial activity against nine clinical isolates revealed that *Enterococcus faecium* and three methicillin-resistant *Staphylococcus aureus* (MRSA) isolates were inhibited by both marine strains. Moreover, *Streptomyces* sp. SWA CA P3.9 reduced the growth of an isolate belonging to *Enterobacter asburiae*, but did not completely inhibit it (Table 2 and Supplementary Figure S5).

## 4. Discussion

Over the last two decades, several culture-independent studies [20,21,22,23,24] have described the taxonomic and metabolic diversity of microbial communities inhabiting the euxinic water layer and the oxic/anoxic interface of the Black Sea. These investigations have led to the discovery of various taxa of anaerobic bacteria and archaea with highly diverse chemotrophic pathways, contributing to a better understanding of microbial adaptations to anoxic and sulfidic niches. Meanwhile, to date, limited investigations of microorganisms inhabiting the Black Sea oxic zone (about 0 to 50 m depth) were carried out [18,19,20]. A recent study [20] reported that microbial communities at 5–30 m depth along the Bulgarian coast were similar to those described in other marine photic zones (e.g., Mediterranean Sea), where Alphaproteobacteria (e.g., *Planktomarina*, *Reyranella*), Gammaproteobacteria (e.g., *Luminiphilus*, *Litoricola*, *Nevskia*), and Cyanobacteria (*Synechococcus*) represented the most abundant groups (>70% of the community). Jaiani and collaborators [19] have evidenced temporal and spatial variations in the composition of microbial communities inhabiting Georgian littoral areas. Overall, their data indicated the dominance of microbial communities by various genera belonging to Cyanobacteria, Alphaproteobacteria, Gammaproteobacteria, Bacilli, and Actinobacteria classes. Comparatively, in the current study, representatives of the Gammaproteobacteria (e.g., *Aliivibrio*), Alphaproteobacteria (e.g., *Planktomarina*), and Flavobacteriia (e.g., NS5 clade) accounted for more than 80% of the bacterial community from the Romanian littoral zone at the Black Sea. These classes generally dominate the photic layer of marine environments due to the photoheterotrophic lifestyle of many of their members [20]. For instance, the genome of *Planktomarina temperata* contains the entire operon responsible for aerobic anoxygenic photosynthesis [47] and thus this species can take advantage of light energy to dominate in environments with low levels of organic nutrients. Although our findings regarding the prevalence of Gammaproteobacteria and Alphaproteobacteria members in the investigated locations were in accordance with those reported in previous studies carried out in other areas of the Black Sea [19,20], Cyanobacteria members (e.g., *Synechococcus* and *Planktothrix*) were much less abundant in the present study (0.4% of the bacterial community), most likely due to the seasonal variations.

In comparison with the total bacterial community that comprised representatives of 185 genera, 46 classes, and 24 phyla, the isolated bacterial strains, which were assigned to 33 genera, 6 classes, and 4 phyla, covered a ~6-fold lower taxonomic diversity. It is a long-standing observation that only a very small fraction (0.001–1%) of environmental microbial assemblages can be cultured on conventional media [48]. At class level, most strains isolated in the current study belonged to the Gammaproteobacteria and Flavobacteriia, which was in accordance with metagenomic data determined in Eforie Nord area. Meanwhile, although a relatively high number of ASVs were assigned to Alphaproteobacteria (Figure 3B), only a small proportion of cultured strains belonged to this class. This observation was also reported in other previous studies carried out in the English Channel [48,49]. At genus level, the isolated strains had a relatively low representativity in the metagenomic community. Among the most abundant taxa were *Marinomonas* and *Pseudoalteromonas* members, which constituted 2.6% and 1% of the community, respectively. The other isolated strains belonged to genera that represented less than 0.5% of the whole bacterial community.

According to the World Register of Marine Species [50], the bacterial strains isolated in the present study belonged to species and genera reported in other marine environments around the world. The isolated strains generally grew optimally at relatively low salinities and temperatures, which is usual for marine bacteria [1]. Some exceptions were, however, noted among the members of the class Bacilli, which were more tolerant to higher salt concentrations and higher temperatures compared with representatives of the other classes (Figure 5). Similar growth characteristics (i.e., tolerance of relatively high temperatures and salinities) have been described previously among related strains (e.g., *Salinicoccus hispanicus* [51], *Jeotgalibacillus campisalis* [52], and *Halobacillus* spp. [53]) isolated from hypersaline environments.

The potential of the isolated marine bacterial strains to produce extracellular hydrolases such as proteases, lipases, and carbohydrases may have important biotechnological implications [7]. Considering that microbial extracellular enzymes generally show functional characteristics related to the physicochemical conditions of the habitats of their sources, marine-derived biocatalysts may show valuable properties such as salt tolerance and cold adaptability. Enzymes with such properties have been proposed as more efficient alternatives to mesophilic counterparts for catalyzing various biotechnologically relevant reactions, e.g., in food processing, biofuel production, and cleaning applications [7,11,12]. 

Among the strains isolated in the current study, of particular interest for biotechnological exploitation may be those that produced multiple extracellular hydrolases with high/medium LEA and showed rapid growth rates, such as *Pseudoalteromonas* spp. (SWA EN P2.3, SWA CA P1.16, MA CA P1.8 and MA CA P1.5) and *Flavobacterium* spp. (SWA EN P2.6 and SWA CA P2.5) (Figure 5). Although most of the retrieved marine strains were closely related (i.e., >99% identity of the partial 16S rDNA sequence) to previously described species (Supplementary Table S1), their extracellular hydrolytic profiles were different (Table 3). For instance, strains SWA CA P1.16 and MA CA P1.5 showed high/medium lipolytic, amylolytic, cellulolytic, and xylanolytic activities, in contrast with the closely related (>99.3% identity) *Pseudoalteromonas arctica* A 37-1-2^T^ strain isolated from the Arctic [54] that produced only protease, lipase, and pectinase. Moreover, strains SWA EN P2.6 and SWA CA P2.5 exhibited five different hydrolytic activities with high/medium LEA, while the homologous (>97.5% identity) strain *Flavobacterium jumunjinense* HME7102^T^ isolated from a coastal lagoon in Korea [55] produced only proteases (Table 3). These results confirm the potential of the Black Sea strains isolated in this study as promising sources of hydrolytic enzymes for various biotechnological applications.

In addition to extracellular enzymes, *Aquimarina muelleri* SWA EN P3.6 and *Streptomyces* sp. SWA CA P3.9 were able to produce antimicrobial compounds with activity against Gram-positive bacterial species *S. aureus* (including MRSA), *L. monocytogenes*, and *E. faecium* responsible for human infections. While the potential of *Streptomyces* spp. to produce efficient antibiotics (e.g., streptomycin and vancomycin) has been widely investigated for more than eight decades [61], the antimicrobial activity of *Aquimarina* spp. has been described in only a few studies conducted over the past nine years [62,63,64,65]. Species of the *Aquimarina* genus, which are considered rare members of the marine biosphere [63], have been described as sources of novel bioactive compounds (i.e., polyketide cuniculene [64] and non-ribosomal peptides/aquimarins [65]) able to inhibit the growth of various marine bacterial species (e.g., *Vibrio* spp.), as well as human pathogens such as MRSA, *Enterococcus faecalis*, *Mycobacterium tuberculosis*, *Acinetobacter baumannii*, and *Candida glabrata* [63,65]. Hence, the antimicrobial activity of the strain SWA EN P3.6 against *S. aureus* (including MRSA) was in accordance with other *Aquimarina* strains. In addition, the strain isolated in the current study was also active against *E. faecium*, representing another life-threatening human pathogen that has acquired resistance to conventional antibiotics [66]. Moreover, considering the rich repertoire of secondary metabolite biosynthetic gene clusters identified in *Aquimarina* spp. genomes [63,65], further investigations on the Black Sea isolate may lead to the discovery of several novel antimicrobial compounds of pharmaceutical interest.

## 5. Conclusions

In search of novel microbial sources of biotechnologically valuable compounds, the taxonomic profile of the uncultured bacterial community inhabiting an underexplored seashore area of the Black Sea was determined, and a marine bacterial strain collection able to produce extracellular hydrolases and antimicrobials was obtained as a first step in exploiting the applicative potential of the bacterial reservoir from this habitat.

A total of 66 bacterial strains were isolated from seawater and assigned to different species and genera commonly found in marine environments. While the cultured taxa belonged to the classes Alphaproteobacteria, Betaproteobacteria, Gammaproteobacteria, Bacilli, Flavobacteriia, and Actinobacteria, the metagenomic bacterial community was ~6 times more diversified and was dominated by representatives of the Gammaproteobacteria, Alphaproteobacteria, and Flavobacteriia. An important fraction of the isolated strains produced extracellular proteases, lipases, and carbohydrases that may be of particular interest for biotechnological applications that require catalysts with marine-related properties, such as salt tolerance and cold adaptability. In addition, two strains belonging to *Streptomyces* and *Aquimarina* genera showed antimicrobial activity against human pathogenic bacterial species, thus being considered potential sources of novel compounds of pharmaceutical interest.

Based on these findings, further investigations can be directed toward the improvement of the cultivability of specific Alphaproteobacteria and Actinobacteria members identified in the marine microbiome as promising sources of metabolites for biotechnologies. Furthermore, the biochemical properties of extracellular enzymes and antimicrobial compounds produced by the newly isolated bacterial strains can be characterized in detail in order to establish their potential biotechnological advantages over currently exploited microbial metabolites.

## Figures and Tables

**Table 1:** Physicochemical parameters of the Black Sea water from EN and CA areas.

Locations	Temperature (°C)	pH	DO (mg·L^−1^)	Salinity (g·kg^−1^)
EN	9.3 ± 0.2	8.1 ± 0.1	4.8 ± 1	18.01 ± 0.08
CA	11.3 ± 0.4	8.1 ± 0.1	9.9 ± 0.9	17.9 ± 0.14

Calculated mean and standard deviation values were obtained from three replicates (sites) for each location; dissolved oxygen (DO).

**Table 2:** Antimicrobial activity of marine bacterial strains against human pathogens ^1^.

		*Aquimarina muelleri* SWA EN P3.6	*Streptomyces* sp. SWA CA P3.9
**Reference strains**	*S. aureus* ATCC 25923	+	+
	*E. coli* ATCC 25922	−	−
	*P. aeruginosa* ATCC 15442	−	−
	*S. enterica* ATCC 14028	−	−
	*L. monocytogenes* ATCC 1911	+	+
**Clinical isolates**	MRSA 388	+	+
	MRSA S1	+	+
	MRSA F1	+	+
	*Acinetobacter* sp. 19047 CNE3	−	−
	*Enterobacter asburiae* 19069 ONE1	−	w+
	*Enterobacter cloacae* 19069 ONE2	−	−
	*Enterococcus faecium* 19040 E1	+	+
	*Klebsiella* sp. 19094 CK1	−	−
	*P*. *aeruginosa* 19053 CNE5	−	−

^1^ Symbols and abbreviations: +: growth inhibition; −: no inhibition; w: weak inhibition; MRSA: methicillin-resistant *Staphylococcus aureus*.

**Table 3:** Extracellular hydrolytic activities of some of the bacterial strains isolated in the present study and of the related type strains ^1^.

Genus	Species	Strain	Hydrolysis of:						Ref.
			Casein	Tween 80	Starch	CMC	Xylan	Pectin	
*Paraglaciecola*	*mesophila*	SWA EN P3.5	-	+	+	+	-	+	This study
		MA CA P3.7	+	+	+	+	−	−	This study
		KMM 241^T^	−	+	+	nd	nd	nd	[56]
*Pseudoalteromonas*	sp.	SWA CA P1.16	−	+	+	+	+	+	This study
	sp.	MA CA P1.5	+	+	+	+	+	−	This study
	*arctica*	A 37-1-2^T^	+	+	− ^a^	− ^b^	−	+	[54]
*Polaribacter*		SWA EN P2.1	+	+	+	+	+	−	This study
	*staleyi*	SWA CA P1.18	+	+	+	+	+	+	This study
		10Alg 139^T^	−	+	+	−	nd	nd	[57]
*Aquimarina*	*muelleri*	SWA EN P3.6	+	+	+	+	−	−	This study
		KMM 6020^T^	+	+	+	−	nd	nd	[58]
*Cellulophaga*	*baltica*	SWA EN P1.16	−	+	−	+	+	−	This study
		SWA CA P1.23	+	+	−	+	+	−	This study
		NN015840^T^	+	−	+	+	nd	nd	[59]
*Flavobacterium*	sp.	SWA EN P2.6	+	+	+	+	+	−	This study
	sp.	SWA CA P2.5	+	+	+	+	+	−	This study
	*jumunjinense*	HME7102^T^	+	−	−	−	nd	nd	[55]
*Isoptericola*	sp.	MA EN P3.8	+	+	+	+	+	+	This study
	*halotolerans*	YIM 70177^T^	−	−	−	nd	nd	nd	[60]

^1^ Symbols and abbreviations: +: positive; −: negative; nd: not determined; Ref.: reference; CMC: carboxymethyl cellulose; ^a^ amylase production was tested on AZCL-amylose; ^b^ cellulase production was tested on AZCL-HE-cellulose.

## Data Availability

The partial 16S rRNA gene sequences of cultured bacterial strains were deposited in GenBank (NCBI) under the accession numbers OL672332–OL672377, OL662943–OL662984 and ON382270. The raw Illumina-derived sequences were deposited in the NCBI Sequence Read Archive under the BioProject: PRJNA875633.

## Supplementary Materials

The following supporting information can be downloaded at: https://www.mdpi.com/article/10.3390/microorganisms10122468/s1, Table S1. Identification of the bacterial strains isolated from the Black Sea; Table S2. Number of reads, ASVs, and alpha diversity indices of bacterial communities from Eforie Nord (EN) sampling sites; Table S3. Number of bacterial isolates able to produce hydrolytic enzymes; Figure S1. The number of shared and unique ASVs (A) and taxa (B) composing the uncultured bacterial communities from the two sampling sites in Eforie Nord (EN1 and EN2); Figure S2. Venn diagrams of the bacterial isolates recovered from Eforie Nord (EN) and Cap Aurora (CA); Figure S3. Screening for extracellular hydrolases using agar plate-based assays (selected photos); Figure S4. The number of bacterial isolates, recovered from Eforie Nord (EN) and Cap Aurora (CA), that produced a particular extracellular hydrolytic enzyme; Figure S5. Antibacterial activities of selected marine isolates against clinical pathogens.
