# Supplementary material for: Bioprospecting for Novel Bacterial Sources of Hydrolytic Enzymes and Antimicrobials in the Romanian Littoral Zone of the Black Sea
Source: Microorganisms. 2022 Dec 14;10(12):2468. doi: 10.3390/microorganisms10122468 (PMC9780896; doi:10.3390/microorganisms10122468)
Supplement: Supplementary file 1 [file microorganisms-10-02468-s001.zip › microorganisms-2071855-supplementary.pdf]

SUPPLEMENTARY MATERIAL

Bioprospecting for Novel Bacterial Sources of Hydrolytic Enzymes and Antimicrobials in the Romanian Littoral Zone of the Black Sea

Robert Ruginescu [1,*], Paris Lavin [2], Lavinia Iancu [1,3], Selma Menabit [1,4] and Cristina Purcarea [1]

[1] Department of Microbiology, Institute of Biology Bucharest of the Romanian Academy, 296 Splaiul Independentei, 060031 Bucharest, Romania
[2] Departamento de Biotecnología, Facultad de Ciencias del Mar y Recursos Biológicos, Universidad de Antofagasta, 601 Angamos Av., Antofagasta, 1240000, Chile
[3] Forensic Science Program, Department of Criminal Justice, University of North Dakota, Grand Forks, ND, 58202, USA
[4] National Institute for Research and Development on Marine Geology and Geoecology-GeoEcoMar, 024053 Bucharest, Romania

*Correspondence: robert.ruginescu@ibiol.ro

Supplementary Table S1. Identification of the bacterial strains isolated from the Black Sea.

Strains code	16S rRNA Gene Sequence			
	Closest relative	Similarity (%)	Size (bp)	GenBank accession no.
**Class Alphaproteobacteria**				
SWA EN P1.14	*Ahrensia kielensis*	99.9	1148	OL672348
SWA CA P1.21	*Ahrensia kielensis*	99.8	1214	OL662950
MA EN P3.9	*Sulfitobacter guttiformis*	99.5	1131	OL672363
MA CA P3.3	*Sulfitobacter guttiformis*	99.4	1107	OL662982
MA CA P2.3	*Litoreibacter* sp.	99.9	1204	OL662961
SWA CA P1.10	*Roseobacter* sp.	98.2	1262	OL662952
**Class Betaproteobacteria**				
SWA CA P1.8	*Hydrogenophaga crassostreae*	99.6	1191	OL662981
SWA CA P2.10	*Hydrogenophaga* sp.	98.6	1211	OL662967
**Class Gammaproteobacteria**				
SWA EN P1.5	*Shewanella aestuarii*	99.0	1109	OL672336
SWA CA P1.1	*Shewanella algicola*	99.3	1198	OL662946
SWA CA P1.20	*Shewanella* sp.	99.1	1006	OL662944
SWA EN P1.1	*Enterovibrio calviensis*	97.5	1145	OL672337
MA EN P2.7	*Enterovibrio calviensis*	98.2	1122	OL672375
MA CA P3.6	*Enterovibrio calviensis*	98.6	1197	OL662971
SWA EN P2.4	*Pseudomonas* sp.	99.5	1164	OL672339
MA EN P1.3	*Pseudomonas* sp.	99.7	1104	OL672361
MA EN P2.10	*Marinomonas rhizomae*	99.1	1097	OL672344
SWA EN P3.16	*Marinomonas primoryensis*	98.9	1113	OL672364
MA EN P3.3	*Marinomonas* sp.	97.7	945	ON382270
SWA CA P1.15	*Marinomonas* sp.	96.3	1261	OL662968
SWA EN P3.5	*Paraglaciecola mesophila*	99.5	1008	OL672347
MA CA P3.7	*Paraglaciecola mesophila*	99.5	1181	OL662964
SWA EN P3.9	*Leucothrix mucor*	97.5	1238	OL672349
SWA CA P2.2	*Leucothrix mucor*	99.6	1210	OL662973
MA EN P2.4	*Pseudoalteromonas neustonica*	99.9	1121	OL672366
SWA EN P1.7	*Pseudoalteromonas marina*	99.6	1089	OL672370
SWA EN P2.3	*Pseudoalteromonas* sp.	99.7	1095	OL672371
SWA CA P1.16	*Pseudoalteromonas* sp.	99.3	1330	OL662947
SWA CA P1.4	*Pseudoalteromonas* sp.	100	1181	OL662956
MA CA P1.8	*Pseudoalteromonas* sp.	99.9	1154	OL662962
MA CA P1.5	*Pseudoalteromonas* sp.	99.6	1346	OL662978
MA EN P1.9	*Granulosicoccus* sp.	99.2	1414	OL672376
MA CA P3.4	*Granulosicoccus* sp.	98.9	1208	OL662983
MA CA P3.1	*Psychrobacter* sp.	99.8	1242	OL662963
SWA CA P3.5-2	*Marinobacter lipolyticus*	98.7	1151	OL662945

**Class Flavobacteriia**				
SWA EN P2.1	*Polaribacter staleyi*	99.8	1099	OL672359
SWA CA P1.18	*Polaribacter staleyi*	99.5	1342	OL662951
SWA EN P3.1	*Aquimarina intermedia*	99.8	1099	OL672350
SWA EN P3.6	*Aquimarina muelleri*	98.5	1095	OL672355
SWA EN P1.16	*Cellulophaga baltica*	99.7	1087	OL672368
SWA CA P1.23	*Cellulophaga baltica*	99.7	1144	OL662975
SWA EN P1.9-1	*Cellulophaga fucicola*	99.9	1191	OL672369
SWA CA P2.6	*Cellulophaga fucicola*	99.9	1186	OL662953
MA EN P3.4	*Zobellia* sp.	99.9	1343	OL672357
SWA EN P1.8	*Zobellia amurskyensis*	99.9	1166	OL672360
SWA CA P1.22	*Zobellia russellii*	100	1208	OL662977
SWA CA P2.1	*Zobellia* sp.	99.9	1191	OL662976
SWA EN P2.6	*Flavobacterium* sp.	97.5	1327	OL672351
SWA CA P2.5	*Flavobacterium* sp.	98.4	1149	OL662969
SWA CA P1.11	*Wenyingzhuangia aestuarii*	99.1	1226	OL662966
SWA CA P2.4	*Maribacter* sp.	99.4	1196	OL662970
MA CA P1.3	*Algibacter* sp.	99.1	1242	OL662974
**Class Bacilli**				
SWA EN P3.4	*Salinicoccus hispanicus*	98.3	1197	OL672332
SWA CA P1.17	*Salinicoccus hispanicus*	98.6	1123	OL662955
SWA EN P3.10	*Bacillus* sp.	99.7	1020	OL672335
MA EN P1.4	*Bacillus* sp.	99.9	1114	OL672362
SWA EN P2.7	*Bacillus* sp.	99.8	1112	OL672338
SWA CA P1.19	*Bacillus* sp.	100	1102	OL662954
SWA EN P1.17	*Metabacillus indicus*	99.6	1136	OL672341
MA EN P2.12-2	*Jeotgalibacillus campisalis*	99.4	1168	OL672342
MA EN P2.6	*Peribacillus simplex*	99.9	1128	OL672374
MA EN P2.14	*Halobacillus* sp.	99.7	1381	OL672346
**Class Actinobacteria**				
SWA EN P3.3	*Micrococcus antarcticus*	99.4	1139	OL672340
MA EN P3.8	*Isoptericola halotolerans*	99.6	1351	OL672343
MA EN P2.5	*Salinibacterium amurskyense*	99.8	1177	OL672372
SWA CA P3.9	*Streptomyces* sp.	99.3	1194	OL662943

Supplementary Table S2. Number of reads, ASVs, and alpha diversity indices of bacterial communities from Eforie Nord (EN) sampling sites.

Sample	Reads	Unique ASVs	Alpha diversity indices			
			Shannon	Simpson	S	Chao1
EN1	349,460	1328	6.521 ± 0.,011	0.9977 ± 0.00009	1328 ± 5	1328 ± 18
EN2	232,300	1395	6.766 ± 0.011	0.9982 ± 0.00009	1395 ± 2	1395 ± 5

Amplicon Sequence Variants (ASVs)

Supplementary Table S3. Number of bacterial isolates able to produce hydrolytic enzymes.

**Class** (No.)	**Protease** No.	**Lipase** No.	**Amylase** No.	**Cellulase** No.	**Xylanase** No.	**Pectinase** No.
Alphaproteobacteria (6)	0	3	0	0	0	0
Betaproteobacteria (2)	0	0	0	0	0	0
Gammaproteobacteria (27)	8	22	10	7	4	4
Flavobacteriia (17)	10	11	9	11	6	1
Bacilli (10)	9	3	9	2	1	6
Actinobacteria (4)	2	2	2	2	2	3

Number of isolates (No.)

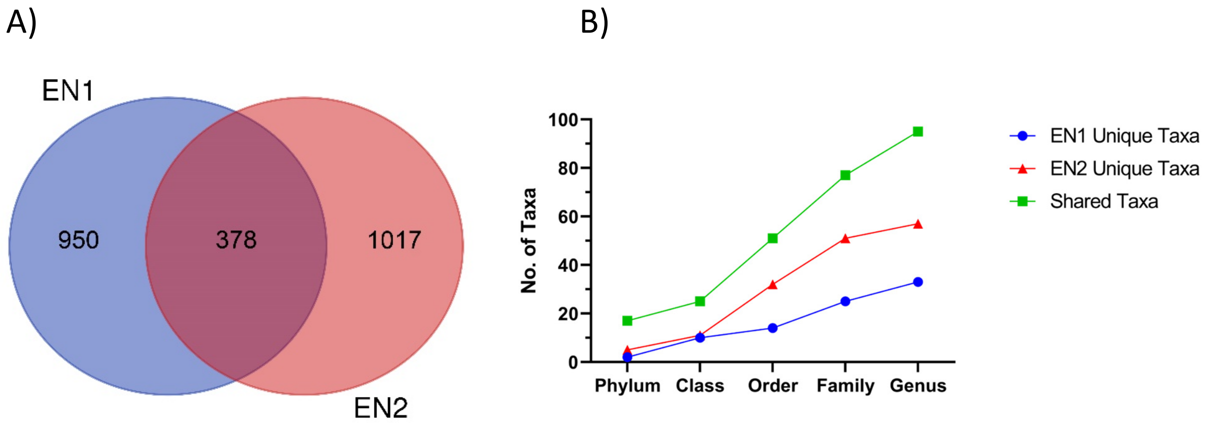

Supplementary Figure S1. The number of shared and unique ASVs (**A**) and taxa (**B**) composing the uncultured bacterial communities from the two sampling sites in Eforie Nord (EN1 and EN2).

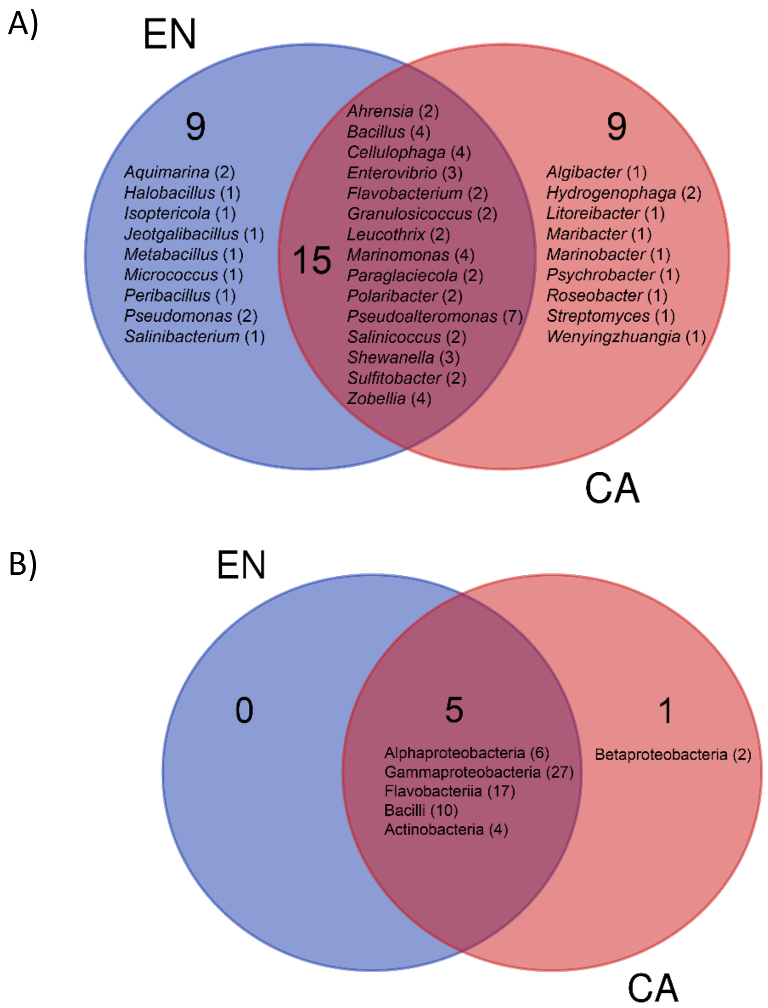

Supplementary Figure S2. Venn diagrams of the bacterial isolates recovered from Eforie Nord (EN) and Cap Aurora (CA). The number of distinct and shared bacterial genera (A) and classes (B) between the two investigated locations were indicated. The number of strains was given between brackets.

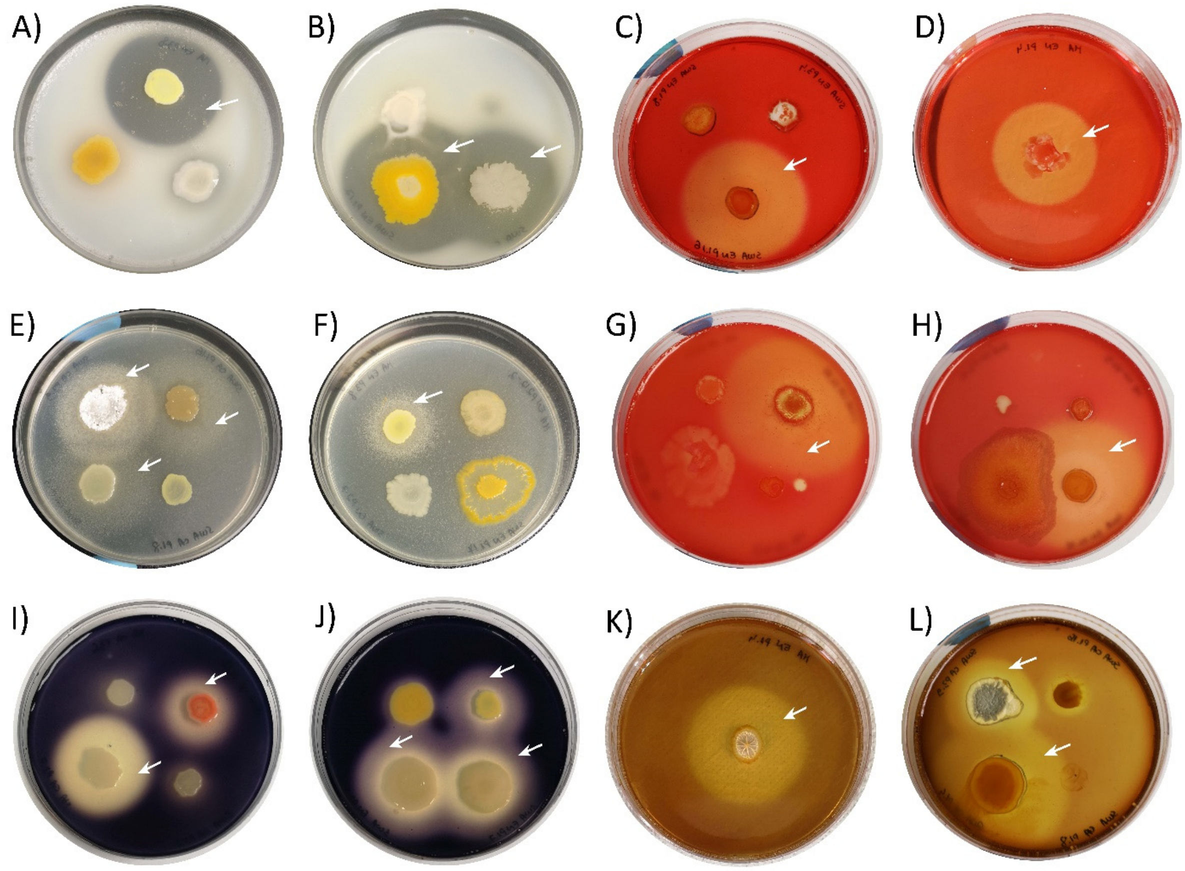

Supplementary Figure S3. Screening for extracellular hydrolases using agar plate-based assays (selected photos). Hydrolysis of casein (**A,B**), CMC (**C,D**), Tween 80 (**E,F**), xylan (**G,H**), starch (**I,J**) and pectin (**K,L**) was indicated by clear or opaque halos (white arrows) around the bacterial spots.

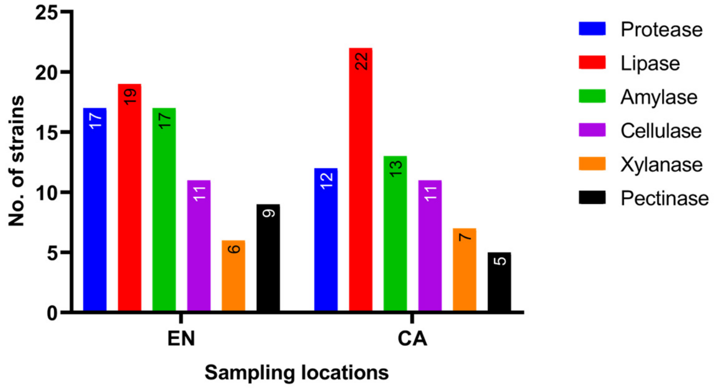

Supplementary Figure S4. The number of bacterial isolates—recovered from Eforie Nord (EN) and Cap Aurora (CA)—that produced a particular extracellular hydrolytic enzyme.

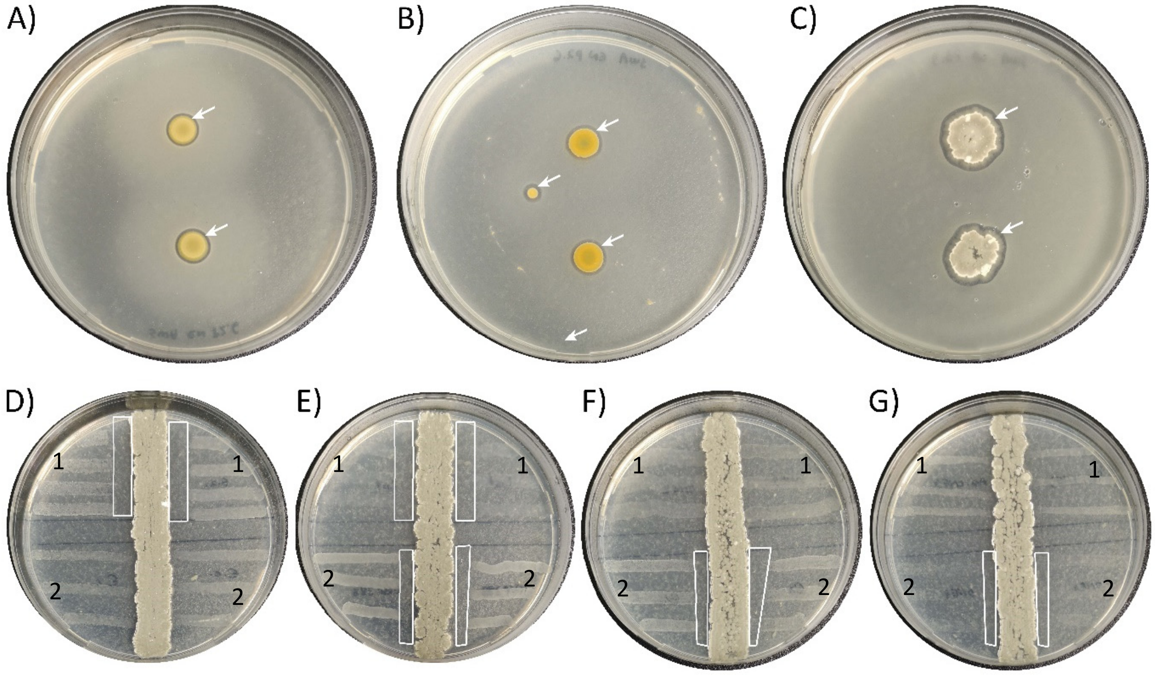

Supplementary Figure S5. Antibacterial activities of selected marine isolates against clinical pathogens. The screening was performed using the soft-agar overlay technique (**A–C**) and the cross-streak method (**D–G**). *Aquimarina muelleri* SWA EN P3.6 against methicillin-resistant *Staphylococcus aureus* S1 (**A**) and *Listeria monocytogenes* (**B**). *Streptomyces* sp. SWA CA P3.9 against methicillin-resistant *S. aureus* S1 (**C**). *Streptomyces* sp. SWA CA P3.9 against *S. aureus* (**D1**), *Escherichia coli* (**D2**), *L. monocytogenes* (**E1**), methicillin-resistant *S. aureus* 388 (**E2**), *Enterobacter cloacae* ONE2 (**F1**), *Enterococcus faecium* E1 (**F2**), *Acinetobacter* sp. CNE3 (**G1**) and *Enterobacter asburiae* ONE1 (**G2**). White arrows/rectangles indicate the areas where the growth of the indicator strains was inhibited.
